# Social Support, Sense of Belonging, and Communication Technology Use Among Paid and Unpaid Caregivers of Middle-Aged and Older Adults

**DOI:** 10.3389/fpubh.2022.898042

**Published:** 2022-05-30

**Authors:** Shinduk Lee, Marcia G. Ory, Deborah Vollmer Dahlke, Matthew Lee Smith

**Affiliations:** ^1^Division of Health Systems and Community-Based Care, College of Nursing, University of Utah, Salt Lake City, UT, United States; ^2^Department of Environmental and Occupational Health, School of Public Health, Texas A&M University, College Station, TX, United States; ^3^Center for Population Health and Aging, Texas A&M University, College Station, TX, United States; ^4^DVD Associates LLC, Austin, TX, United States

**Keywords:** communication technology, social support, sense of belonging, caregiving, older adult

## Abstract

**Objectives:**

The objectives of this study are to: (1) describe communication technology use among paid and unpaid middle-aged and older caregivers of adults 50 and older in a natural (non-experimental) setting; and (2) examine the association between communication technology use, perceived social support, and sense of belonging in this population.

**Methods:**

Means and standard deviations, or frequencies and percentages, were used to describe study participants. Chi-square tests or independent sample *t*-tests were used to compare sociodemographic characteristics, communication technology use, perceived social support, and sense of belonging to the local community between paid and unpaid caregivers. Multivariable regression analysis was performed to predict each outcome (i.e., sense of belonging and social support) based on the use of texting or communication applications.

**Results:**

The average age of participants was age 64.2 years, and the majority was female (74.8%) and non-Hispanic White (66.9%). Compared to paid caregivers, unpaid caregivers were older (64.5 vs. 62.2 years, *p* = 0.022) and a larger proportion were non-Hispanic White (70.8% vs. 47.7%, *p* < 0.001). Nearly 83% of the study participants reported using texting or communication applications (81.5% among paid caregivers and 83.1% among unpaid caregivers, *p* = 0.718). After adjusting for caregivers' age, sex, race/ethnicity, and education, a significantly higher sense of belonging was observed among paid caregivers than unpaid caregivers (*b* = 9.40, *p* = 0.009). After adjusting for caregivers' age, sex, race/ethnicity, and education, the use of texting or other communication applications significantly increased caregivers' perceived availability of social support (*b* = 0.35, *p* = 001).

**Conclusions:**

These study results showed a greater sense of belonging to the local community among paid caregivers compared to unpaid caregivers. The use of communication technology was associated with an increased sense of belonging to their local community among paid caregivers, yet the use of communication technology did not contribute to feelings of belonging among unpaid caregivers. In an aging society, both paid and unpaid caregivers are essential elements of the care system. Research is needed to understand the social support needs of paid and unpaid caregivers and the types of interventions to promote social support and community engagement for both groups.

## Introduction

Over 40 million Americans are estimated to provide unpaid care to a family member or friend aged 50 years or older in 2020 (1). On average, unpaid caregivers for older adults provide over 22 weekly hours of care, and they assist their care recipients with basic and instrumental activities of daily living and medical tasks, as needed (1). Consistent evidence shows that informal caregivers have reduced social activities over time, which increases caregiver burden and negatively influences caregivers' health and quality of life (2–4). In addition to unpaid or informal caregivers (e.g., family members and friends), paid caregiving services are increasingly needed and more prevalent among care recipients of older ages.

Through social networks and interactions, caregivers can access social support in the form of emotional, informational, and other tangible and intangible resources. Social support can protect caregivers against feeling burdened (5) by providing resources to eliminate or reduce the perceived stress and alleviate the impact of stressors (6, 7). Having a sense of belonging, which is closely associated with perceived social support (8), can act as a buffer against caregiving burden and protect caregivers' mental and social well-being (9–11). Sense of belonging is described as a “component of connecting one's self into the fabric of surrounding people, places, and things” (12). While feelings of belonging have been investigated among various populations (e.g., young adults, older adults, and patients) (9, 13, 14), limited studies have examined the sense of belonging felt by caregivers. Furthermore, paid and unpaid caregivers may have different needs, preferences, and barriers related to connecting with their community and interpersonal groups; however, limited studies have examined or compared feelings of social support or sense of belonging among paid and unpaid caregivers (15).

Communication technologies can be a useful tool to connect caregivers to their social networks and enable them to access resources (16, 17). Specifically, this study focuses on communication technologies (e.g., texting and virtual communication applications), which are considered promising tools to mitigate social isolation among older adults (18, 19). According to Zwingmann et al. (20), family caregivers' perceived social isolation can be alleviated through caregiver support groups established in a more flexible and private setting, such as telephone- and internet-based communications. However, only 7% of informal caregivers of older adults use the communication technology to connect with other caregivers (1). Little is known about use of communication technology among caregivers, especially among paid caregivers, and its association with their social well-being in a natural (i.e., non-experimental) context. Furthermore, the majority of older adults' caregivers are older adults, and despite the increasing communication technology use among the older adult population, it has been suggested that older adults may only be using a few features of communication technologies (21).

This cross-sectional survey study aims to describe communication technology use among paid and unpaid, middle-aged and older caregivers of adults 50 years and older. This study also examines the relationship between communication technology use and perceived social support and sense of belonging to local communities among paid and unpaid caregivers of middle-aged and older adults. Intuitively, we hypothesized communication technology use to be positively associated with social support and sense of belonging to the local community among both paid and unpaid caregivers by facilitating social interactions and increasing their access to social support. Acknowledging the importance of both paid and unpaid caregivers in the system of care for older adults, this study included both caregiver types. This study further explored potential differences in the association between the communication technology use and social support and sense of belonging between paid and unpaid caregivers. Given the subtle differences in their caregiving contexts (e.g., training and work expectations for technology use), paid and unpaid caregivers may use communication technology differently for connecting with their local community or accessing social support.

## Methods

### Data

This study utilized cross-sectional Qualtrics panel survey data collected from adult caregivers of middle-aged and older adults in November 2019. To be eligible to participate in the survey, one must be 18 years and older, be paid or unpaid caregivers of at least one middle-aged and older adults (50 years and older) who lived in a home environment. Quota sampling was used to ensure inclusion of diversity in the study sample (22). The quotas were applied in terms of regions (Northwest, Midwest, West, and South), gender, age, and race/ethnicity (maximum 60% non-Hispanic White) to resemble the adult caregiver populations. Among the overall respondents (*N* = 626), this study was limited to middle-aged and older caregivers (*n* = 504) because a larger proportion of middle-aged and older adults provide care for adults 50 years in the US, and less is known about communication technology use in this age group, despite its increasing availability uptake. Paid and unpaid caregivers are distinct caregiver populations, and communication technology may play different roles for social support and connecting them with their local communities. This study included both paid and unpaid caregivers to provide greater understanding about the social support needs of both groups. The study was reviewed and approved by the Texas A&M University institutional review board (IRB2019-1128M).

### Measures

Primary outcomes were caregivers' sense of belonging to their local community and perception of social supports available to them. Sense of belonging was examined using a single item (“my sense of belonging to my local community is…”), and participants rated their response on a slider ranging from 0 (none) to 100 (very strong). The survey adapted Lubben's Social Network Scale (23) to assess perceived social support available. Participants were asked how many relatives, friends, neighbors, other than the care recipient they see or hear at least once a month, feel close to, and feel they can call on for help (e.g., chores, transportation). Response options were: none, one, two-to-four, five-to-eight, nine or more, and uncertain. None responded ‘uncertain’ to any of the three items, and hence the response option was removed from the analysis. The Cronbach's alpha for the three items was 0.81.

The primary independent variable of interest was caregivers' use of communication technology (texting or communication applications). Participants were asked if they use texting or communication applications. WhatsApp, Facetime, Skype, and Google Hangouts were given as examples of communication applications. The use of communication technology was not restricted for caregiving purposes or any other purpose to capture the technology use in general.

The effect modifier was caregivers' paid status. Participants self-reported their caregiver type as paid (8.7%, *n* = 44), unpaid (82.9%, *n* = 418), and both paid and unpaid (8.3%, *n* = 42). For this analysis, caregivers who received payment for caregiving (i.e., “paid” and “both paid and unpaid”) were considered as paid caregivers (17.1%, *n* = 86).

Sociodemographic variables examined included were age in years, sex, race/ethnicity, education, household income, perceived financial distress (24), and type of geographic area. Geographic area types were classified into metropolitan and non-metropolitan areas based on self-reported ZIP Codes and Rural-Urban Commuting Area (RUCA) Codes (25). Caregiver context was examined by asking their total weekly hours of caregiving to adults over age 50 living in a home environment and whether their family relationship was strained due to caregiving (yes/no).

### Analyses

Means and standard deviations or frequencies and percentages were used to describe the study participants. Next, Chi-square tests or independent sample *t*-tests were used to compare sociodemographic characteristics, communication technology use, perceived social support, and sense of belonging to the local community between paid and unpaid caregivers. The descriptive statistics and comparison results are presented in Table 1. In addition, Chi-square tests and independent *t*-tests were used to compare sociodemographic characteristics of caregivers who did and did not report using communication technology. These analyses were performed separately for paid and unpaid caregivers. Multivariable regression analysis was performed to predict each outcome (i.e., sense of belonging and social support) based on the use of texting or communication applications. Multivariable regression analyses were repeated for each outcome variable after including the caregivers' paid status (paid/unpaid) and the interaction term between the caregivers' payment status and the use of communication technology. All regression models were adjusted for caregivers' age, sex, race/ethnicity, and education.

**Table 1:** Study participants' sociodemographic characteristics, technology use, sense of belonging to their local community and social bonds, by caregiver payment status (paid/unpaid).

**Characteristics**	**Mean (SD) or Frequency (%)**			***Paid vs. unpaid:*** ***p-value***
	**Overall** **(*N* = 504)**	**Paid** **(*n* = 86)**	**Unpaid** **(*n* = 418)**	
**Sociodemographic characteristics**				
Age (years)	64.2 (8.53)	62.2 (9.34)	64.5 (8.32)	0.022
Female	376 (74.8%)	68 (79.1%)	308 (73.9%)	0.311
Non-Hispanic White	335 (66.9%)	41 (47.7%)	294 (70.8%)	<0.001
High school or lower educational attainment	101 (20.6%)	24 (30.0%)	77 (18.8%)	0.023
Household income < $50,000	247 (50.4%)	47 (58.8%)	200 (48.8%)	0.103
Financial stress				0.366
End up with some money left over	229 (47.6%)	32 (41.6%)	197 (48.8%)	
Have just enough money to make ends meet	180 (37.4%)	30 (39.0%)	150 (37.1%)	
Not have enough money to make ends meet	72 (15.0%)	15 (19.5%)	57 (14.1%)	
Non-metropolitan area	45 (9.1%)	7 (8.6%)	38 (9.2%)	0.868
**Caregiving context**				
Weekly hours of caregiving for adults over 50 years	53.5 (50.14)	45.7 (38.33)	54.9 (52.20)	0.123
**Technology use**				
Texting or communication applications	411 (82.9%)	66 (81.5%)	345 (83.1%)	0.718
**Sense of belonging** (score 0–100, higher score = stronger sense of belonging)	56.7 (29.16)	65.1 (30.94)	55.1 (28.6)	0.005
**Social support availability** (score 1–5, higher score = more social support)	2.7 (0.88)	2.7 (0.94)	2.7 (0.87)	0.838

## Results

### Study Participants

The average age of participants was 64.2 years (standard deviation = 8.53) and the majority was female (74.8%) and non-Hispanic White (66.9%). Compared to paid caregivers, unpaid caregivers were significantly older (64.5 vs. 62.2 years, *p* = 0.022) and a larger proportion were non-Hispanic White (70.8 vs. 47.7%, *p* < 0.001). Among the study participants, a higher percentage of paid caregivers had lower educational attainment (i.e., high school graduate or less education) than unpaid caregivers (30.0 vs. 18.8%, *p* = 0.023). The average weekly hours of caregiving for adults 50 years and older was 53.5 h (45.7 h among paid caregivers and 54.9 h among unpaid caregivers, *p* = 0.123). Paid caregivers reported a higher sense of belonging to their local community (65.1 vs. 55.1, *p* = 0.005), and both paid and unpaid caregivers reported some social support (i.e., approximately one to four relatives, friends, neighbors, other than their care recipient that they see or hear from at least once a month, feel close to, and feel they can call on for help) (*p* = 0.838).

### Communication Technology Use

Nearly 83% of the study participants reported using texting or communication applications (81.5% among paid caregivers and 83.1% among unpaid caregivers, *p* = 0.718) (Table 1). For both paid and unpaid caregivers, there were no statistically significant differences in sociodemographic characteristics between those who used and did not use communication technologies.

### Sense of Belonging

After adjusting for caregivers' age, sex, race/ethnicity, and education, a significantly higher sense of belonging was observed among paid caregivers than unpaid caregivers (*b* = 9.40, *p* = 0.009) (Table 2). There was no statistically significant difference in sense of belonging based on caregivers' use of texting or communication applications (*p* = 0.218). However, the interaction effect showed that among paid caregivers, the use of texting or communication application significantly increased their sense of belonging, but this relationship was less among unpaid caregivers (b_interaction = 21.28, *p* = 0.022).

**Table 2:** Sense of belonging based on caregivers' payment status and use of communication technologies.

**Predictors**	**Regression coefficient estimate**	**Regression coefficient standard error**	***p*-value**
**Model 1^a^**			
Using texting or communication applications (reference = not using)	4.41	3.57	0.218
Paid for caregiving (reference = not paid for caregiving)	9.40	3.60	0.009
**Model 2^a^**			
Using texting or communication applications (reference = not using)	0.73	3.90	0.853
Paid for caregiving (reference = not paid for caregiving)	−8.25	8.49	0.332
Interaction term (Using texting or communication application X Paid for caregiving)	21.28	9.28	0.022

a *Both models 1 and 2 were adjusted for caregivers' age, sex, race/ethnicity, and education*.

### Social Support

After adjusting for caregivers' age, sex, race/ethnicity, and education, the use of texting or other communication applications significantly increased caregivers' perceived availability of social support (*b* = 0.35, *p* = 001) (Table 3). There was no statistically significant difference in perceived social support availability based on caregivers' payment status (*p* = 0.816). There was no statistically significant difference in the relationship between perceived social support availability and use of texting or communication applications, based on caregivers' payment status (b_interaction = −0.36, *p* = 0.216).

**Table 3:** Social support availability based on caregivers' payment status and use of communication technologies.

**Predictors**	**Regression coefficient estimate**	**Regression coefficient standard error**	***p*-value**
**Model 1^a^**			
Using texting or communication applications (reference = not using)	0.35	0.11	0.001
Paid for caregiving (reference = not paid for caregiving)	0.03	0.11	0.816
**Model 2^a^**			
Using texting or communication applications (reference = not using)	0.41	0.12	0.001
Paid for caregiving (reference = not paid for caregiving)	0.32	0.27	0.223
Interaction term (Using texting or communication application X Paid for caregiving)	−0.36	0.29	0.216

a *Both models 1 and 2 were adjusted for caregivers' age, sex, race/ethnicity, and education*.

## Discussion

This study described communication technology use among middle-aged and older, paid and unpaid caregivers of adults 50 years and older in a natural (non-experimental) setting. It also examined the association between their communication technology use, perceived social support, and sense of belonging. In this cross-sectional online survey study, more than 80% caregivers used some form of communication technology (i.e., texting or other communication applications). This study included caregivers who were age 50 and older, and the observed rate of communication technology use is comparable with the rates reported in the 2020 American Association of Retired Persons (AARP) report on older adults' technology use (26). According to the report, about 91% of adults at 50 and older use technology to stay connected with friends and family. About 94% of adults ages 50 and older reported daily use of smartphones, and 83% of those who own a smartphone used instant messaging or email applications. It is important to note that both the current study and AARP report used online surveys, and therefore, the communication technology use rates may be overestimated. Despite the likelihood of over-estimation, this current study result supports high communication technology use rates among middle-aged and older caregivers and suggests that communication technology-based interventions may be useful to promote or maintain social relations and activities among middle-aged and older caregivers of middle-aged and older care recipients.

In the current study, paid caregivers reported a greater sense of belonging to their local community compared to unpaid caregivers. Communication technology use was positively associated with a sense of belonging to their local community among paid caregivers; however, this association was less pronounced among unpaid caregivers. According to Hagerty et al., antecedents of a sense of belonging are an individual's energy for involvement, likelihood, and willingness to be involved, and the likelihood of shared or complementary characteristics (12). In 2020, about 62% of informal caregivers of middle-aged and older adults were employed, and about 60% of them worked full-time (1). Unpaid caregivers, who might have another job, might have lower energy in the caregiving role and/or opportunity to be involved in the local community than paid caregivers. In terms of Hagerty's conceptualization of sense of belonging, paid caregivers may have more energy, and shared complementary characteristics with other paid caregivers in the similar field of occupation. Given the nature of paid caregiving, these caregivers may belong to other networks of employees or professional associations, where posing questions, posting feelings, and offering support on communication technologies may be more commonplace (or even expected). While the use of communication technology can potentially facilitate an individual's involvement in their local community, it may not be solely sufficient to promote involvement in the local community in those who lack the antecedents (i.e., energy, willingness, and shared or complementary characteristics).

In line with the study hypothesis, this study indicates a positive correlation between communication technology use and social support. This finding confirms prior work and strengthens the evidence that communication technology is a promising tool to enhance perceived social support among middle-aged and older caregivers (27, 28). In that there was no statistically significant difference in this relationship between paid and unpaid caregivers, communication technology-based interventions targeting social support can be effective for paid and unpaid caregivers.

This study has some limitations. First, this study used an online panel survey, and the study sample may not be representative of the middle-aged and older caregiver population in the US. Despite our efforts to resemble the middle-aged and older caregiver population, the nature of data collection (i.e., *via* online) excluded those without access to internet (22). According to the 2020 AARP report on family caregivers of adults 50 years and older, the majority of caregivers of adults 50 years and older were 50 years and older (56%) and non-Hispanic White (61%), and about 31% had high school graduate or less education (NAC and AARP). Using the data from the Integrated Public Use Microdata Series (IPUMS), the Paraprofessional healthcare Institute (PHI) reported that about 66% of the home care workers were younger than 55 years old; about 37% were non-Hispanic White; and about 53% had high school graduate or less education (29). While the reports do not specifically describe the sociodemographic characteristics of the caregivers 50 years and older, the observed sociodemographic comparison between paid and unpaid caregivers in this was comparable to the national reports. In this study, compared to paid caregivers, unpaid caregivers were older, were more likely to be a non-Hispanic White individual, and were less likely to have lower educational attainment (i.e., high school graduate or less education). To complement these findings, future efforts should utilize diverse sampling and data collection methods to advance what is known about communication technology use among paid and unpaid caregivers. Second, while the study sample included paid caregivers, the number was small (*n* = 86, 17%) and may not be representative of all paid caregivers based on their specialty or employer type. Furthermore, caregivers' paid status was loosely defined based on self-reported data (i.e., “what type of caregiver are you?” and “For this person, the oldest person for whom you provide caregiving, are you a paid caregiver?”). Third, this study examined only specific categories of communication technology (i.e., texting and other instant messaging and audio and video calls). While text message is the most frequently used technologies by middle-aged and older adults to stay connected with their social networks (92% in both 2019 and 2020), the online survey excluded the use of email and social media, which are also communication technology used to connect older adults to their networks (21, 26). Further, measurement of communication technology use did not distinguish between specific uses for professional or personal reasons among paid caregivers. Future studies should incorporate additional items about the types and purposes of communication technology used among paid and unpaid caregivers. Fourth, the phrase “local community” was not defined for the survey respondents, which may have introduced subjectivity and bias in its interpretation across populations.

Despite these study limitations, the study provides new insights about the use of communication technology among paid and unpaid, middle-aged and older caregivers of adults 50 years and older. Sense of belonging (12, 30) and social support are important constructs related to health, and this study suggests positive relationships between these two constructs and communication technology use among these caregivers. Findings provide evidence supporting the potential of communication technology-based interventions to promote a sense of belonging and social support among middle-aged and older caregivers. Furthermore, this study also shows that communication technology may play different roles in connecting middle-aged and older caregivers to their local communities, which may also differ for paid and unpaid caregivers. Given the observed differences between these caregiver groups, a diverse set of interventions are likely needed to enhance sense of belonging and social support across paid and unpaid caregiver populations. For paid caregivers, such interventions may be dictated by the caregivers' training, issued technology, and industry standards. Interventions for paid caregivers may be more easily assessed in terms of the intervention reach, implementation, and effectiveness, relative to unpaid caregivers. Regardless of the paid or unpaid caregiver audience, interventions including communication technology should take into consideration aspects of the caregivers' social network and their access to, comfort with, and preference for communication technology. Because paid and unpaid caregivers are essential resources supporting an aging society, it is critical to understand their respective needs and the types of interventions that would be feasible and effective to improve their feelings of belonging and support.

## Data Availability Statement

The datasets presented in this article are not readily available because of the language in the original informed consent. The data may be available on request from the co-author, MO, upon IRB approval. Requests to access the datasets should be directed to MO, mory@tamu.edu.

## Ethics Statement

This study involves human participants and was reviewed and approved by Texas A&M University IRB. The participants provided their electronic informed consent to participate in this study.

## Author Contributions

SL led the data analyses and development of the initial manuscript draft. MO contributed to the data analyses and development of the initial manuscript draft. All authors contributed to the study conceptualization, data collection, and revision of the manuscript.

## Funding

The caregiver survey was funded by contributions from the Texas A&M Center for Population Health and Aging, DVD Associates, LLC, and Clairvoyant Networks Incorporated.

## Conflict of Interest

The study was partially supported by Mr. Steve Popovich, the president and CEO of Clairvoyant Network. Clairvoyant Network is a company developing products related to caregiving solutions. Mr. Popovich was not involved in the conceptualization, methodology, analyses, and development of this manuscript. The authors declare that the research was conducted in the absence of any commercial or financial relationships that could be construed as a potential conflict of interest.

## Publisher's Note

All claims expressed in this article are solely those of the authors and do not necessarily represent those of their affiliated organizations, or those of the publisher, the editors and the reviewers. Any product that may be evaluated in this article, or claim that may be made by its manufacturer, is not guaranteed or endorsed by the publisher.
